# A Micron-Sized Laser Photothermal Effect Evaluation System and Method

**DOI:** 10.3390/s21155133

**Published:** 2021-07-29

**Authors:** Jingjing Xu, Ming Zeng, Xin Xu, Junhui Liu, Xinyu Huo, Danhong Han, Zhenhai Wang, Lan Tian

**Affiliations:** 1 Institute of Microelectronics, Shandong University, Jinan 250102, China; xujj@sdu.edu.cn (J.X.); zengming@sdu.edu.cn (M.Z.); xinxu2017@mail.sdu.edu.cn (X.X.); ljunhui@mail.sdu.edu.cn (J.L.); huoxinyu@sd.chinamobile.com (X.H.); 2 Shenzhen Research Institute, Shandong University, Shenzhen 518057, China; 3 Beijing Research Institute of Mechanical Equipment, Beijing 100854, China; handanhong@126.com (D.H.); wzh632@163.com (Z.W.)

**Keywords:** temperature measurement system, photothermal effects, laser-thermocouple interaction, micron-sized laser, wavelength, thermocouples

## Abstract

The photothermal effects of lasers have played an important role in both medical laser applications and the development of cochlear implants with optical stimulation. However, there are few methods to evaluate the thermal effect of micron-sized laser spots interacting with other tissues. Here, we present a multi-wavelength micro-scale laser thermal effect measuring system that has high temporal, spatial and temperature resolutions, and can quantitatively realize evaluations in real time. In this system, with accurate 3D positioning and flexible pulsed laser parameter adjustments, groups of temperature changes are systematically measured when the micron-sized laser spots from six kinds of wavelengths individually irradiate the Pd/Cr thermocouple junction area, and reference data of laser spot thermal effects are obtained. This work develops a stable, reliable and universal tool for quantitatively exploring the thermal effect of micron-sized lasers, and provides basic reference data for research on light-stimulated neuron excitement in the future.

## 1. Introduction

Photothermal effects play a very important role in not only medical treatment, but also light-regulated nerve excitement research [1,2,3]. More and more studies have confirmed that light can directly cause neuronal excitement under non-transgenic conditions. As early as the 1960s, Arvanitaki et al. quantified the effect of visible and near-infrared light with different wavelengths on nerve cells [4]. In 1971, Fork et al. measured nerve impulses without causing obvious irreversible damage to nerve tissue when continuously irradiated by the 488 nm laser, demonstrating the safety of light-stimulated neurons for the first time [5]. In 2002, Hirase et al. used near-infrared pulsed lasers to stimulate the cerebral cortex, and found that the action potentials generated on neurons are likely to be related to the amount of laser radiation [6]. In 2005, Wells et al. in Vanderbilt University revealed that mid-infrared lasers with a wavelength of 2–6 μm can induce action potentials without damage in the sciatic nerves of rats [7]. From 2006 to 2011, Richter’s team at Northwestern University further tried to stimulate the auditory nervous system of guinea pigs with pulsed infrared lasers, and confirmed that it was feasible to stimulate the spiral ganglion cells to induce auditory nerve impulses with pulsed lasers. They also initially explored the influence of laser parameters such as laser wavelengths, pulse width, stimulation frequency and pulse energy [8,9,10,11,12]. Their work created a precedent in the study of cochlear implants with optical stimulation. Many research groups have followed up ear nerve stimulation research with pulsed lasers in various bands [13,14,15,16,17]. The latest research from our team found that a pulsed laser of 450 nm wavelength can cause calcium ion channels on spiral ganglion neurons to open [18]. However, mechanisms of the laser-induced excitement of neurons and non-transgenic spiral ganglion cells are still unclear. The photochemical and photoelectric effects are basically excluded [19]. At present, it is generally accepted that it is the result of individual or a combination of factors of photoacoustic and photothermal effects [20,21]. The photoacoustic effect is stated as a pulsed laser which generates pressure waves in tissues and stimulates the cochlear hair cells [20,22,23]. Studies supporting the photothermal effect have found that the absorption of lasers by nerve tissue caused a temporary local temperature increase in the target tissue, which may activate voltage-gated or heat-sensitive ion channels to generate nerve impulses [10,11,19,24]. In addition, the neural electromechanical soliton model also claims the nerve impulse conduction in the form of heat [25,26,27].

A systematic study of the photothermal response of lasers in various frequency bands is of great significance for clarifying the mechanism in light-induced nerve excitation. At present, research on laser photothermal evaluations is mostly based on the subjective feeling of subjects [28], which is not objective enough; or on the measurement of temperature changes resulting from the photothermal effect of lasers on a centi/millimeter scale [29]. However, studies on light-induced nerve excitement always employ laser beams with micron-sized diameters, and there are a lack of available temperature measuring devices and systems for micron-sized lasers. Firstly, the widely developed radiative measurement methods using fluorescence or infrared light are inapplicable, because the fluorescence probes are easily affected by intracellular chemical components [30,31], and the infrared light used to measure temperature and lasers used for photothermal study will interfere with each other [32], resulting in the inaccuracy of photothermal effect evaluations. Secondly, the micro-scale laser irritation area determines the requirement of micron sizes for temperature sensors. In addition, it is hard to position micron-sized laser beams on a micron-sized sensing region with the naked eye and with manual operation. Moreover, the close irradiation requirements in light-stimulated nerve excitement research limit the introduction of microscopes to assist positioning.

Here, we develop a non-radiative temperature measurement system constructed by a Pd/Cr thin film thermocouple array, 3D positioning module and real-time data processing program, which can be used to quantitatively evaluate the photothermal effect of micron-sized lasers and other tissues. To obtain the background reference data of the system and establish a complete photothermal evaluation model in the future, the temperature response of Pd/Cr thin film thermocouples under the direct radiation of pulsed lasers in the 450–1064 nm band are systematically explored, excluding the other influences from water, protein molecules and other substances.

## 2. Methods

### 2.1. Fabrication of Thermocouple Device

The devices, including thin film thermocouple (TFTC) arrays for measuring temperature, were fabricated via standard cleanroom techniques on 4-inch wafers, as published elsewhere [33,34]. The Si <100> wafers were coated with 400 nm thick Si_3_N_4_, on which the Pd/Cr thermocouple arrays serving as the temperature sensors were constituted by the deposition of Pd thin films and Cr thin films. The freestanding Si_3_N_4_ windows were etched using published methods [29,35] to improve the sensitivity of the thermocouple sensors on them. Later, the wafer with TFTC arrays was electrically connected with the designed printed circuit board using a wire-bonding process.

### 2.2. Data Processing

#### 2.2.1. Δ*T*-*ΔV*

Here, a thermocouple made of Pd and Cr thin films was chosen as the thermometer. Their junction served as the hot end, and their electrode pads served as the cold ends. The voltage difference between the two cold ends was proportional to the temperature difference between the hot junction and the cold ends:

(1) $$\Delta T=\frac{\Delta V}{S}$$

where *S* is the Seebeck coefficient difference between these two metals, Pd and Cr. The thermopower of Pd/Cr thermocouples was calibrated on a homemade calibration platform [36], and showed a stable sensitivity of 21 ± 0.1 μV/K and an accuracy of ± 20 mK at room temperature [37].

#### 2.2.2. The Relationship between the Physical Parameters of the Laser

We calibrated the peak power of the semiconductor laser sources with six different wavelengths, and calculated its average power in pulse mode, $P_{average}$, with the following formula:

(2) $$P_{average}=P_{peak}\times t_{duration}\times f$$

where $P_{peak}$ is the peak power of the pulsed laser, $t_{duration}$ is the duration of a pulse (200 μs in this work), and f is the pulse repetition frequency. The energy of a single pulse, $E_{peak}$, is:

(3) $$E_{single}=P_{peak}\times t_{duration}$$

Additionally, the laser energy density of a pulsed laser irradiated on a device per second, i.e., the average energy density per second, $D_{energy}$, can be calculated with the following formula:

(4) $$D_{energy}=\frac{E_{single}\cdot f}{S_{spot\;area}}$$

where $S_{spot\;area}$ is the laser’s spot area on the device of 0.19 mm^2^ when the laser beam reaches the device 1 mm away.

## 3. Results

### 3.1. The Measurement Method for Evaluating Photothermal Effect of Micron-Sized Lasers

**Figure 1:**
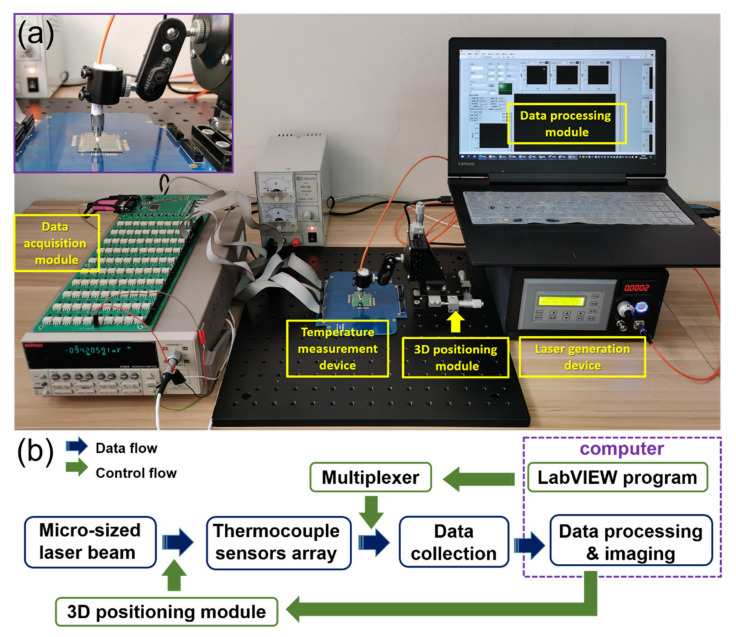
The temperature measurement system for photothermal evaluation of micron-sized lasers. (**a**) The photograph of the working system in research; (**b**) schematic diagram of the working system.

**Figure 2:**
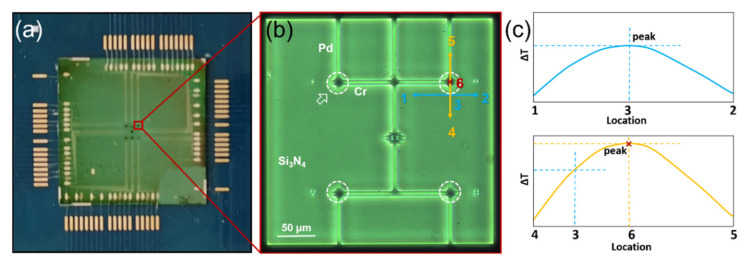
The precise positioning method. (**a**) A diagram of devices for temperature measurement; (**b**) photograph of the Pd/Cr thin film thermocouple array on a Si3N4 freestanding window; (**c**) the voltage output of the thermocouple when fine-tuning the position of laser fiber, in which the upper is related to tuning along blue direction and the lower is along the orange line in (**b**).

The photothermal temperature measurement system for micron-sized laser was composed of four parts: the laser generation device, the three-dimensional positioning module, the temperature measurement device, the data acquisition and processing module, as shown in Figure 1. The laser generator module could output six kinds of lasers, including 450 nm, 525 nm, 638 nm, 810 nm, 980 nm and 1064 nm. The laser output mode (continuous mode or pulse mode), light intensity, as well as the pulse width and repetition frequency in pulse mode, could be adjusted. The movement accuracy of the 3D positioning module could reach 0.03 mm, which helped the laser beam to irradiate the junction area of the thermocouple accurately. The temperature measurement device is made of nine dependent measurement units, on each of which four micron-sized Pd/Cr thin film thermocouples are prepared on the freestanding Si_3_N_4_ platform (400 nm) to improve the sensitivity and accuracy of the measurement, as shown in Figure 2a. Based on the Seebeck effect, thermocouples can convert the temperature difference between the hot and cold ends into a weak voltage between the cold ends of Pd and Cr film arms. The data acquisition and processing module is constituted of nanovoltmeter, multiplexer and LabVIEW software. The output voltage of the Pd/Cr thermocouples was in the order of microvolts; therefore, a high-precision nanovoltmeter, Keithley 2182A, was used to measure the voltage between the ends of the two ends of the thermocouples, and transmit the data to the processing part in LabVIEW software in computer, through GPIB-HS. The introduction of the multiplexer is one of the keys to realizing precise positioning of the laser beam. Through the preset program in LabVIEW, the multiplexer can sequentially turn on the electrical path to each thermocouple to enable the nanovoltmeter to detect its output voltage. Cyclic detection for the four thermocouples needed 0.4 s, because the switching time of the used mechanical switches was almost 80 ms. As a result, the continuous cyclic temperature detection of the thermocouple array was similar to real-time measuring. The LabVIEW program can not only control the data acquisition, but also display the output voltage value of each thermocouple in real time.

In this system, a method for precisely positioning the laser beam was designed by combining the real-time temperature measurement of the thermocouple array shown in Figure 2 and the 3D positioning module. As shown in Figure 2b, the distance between thermocouples was 150 μm, and the laser beam had an emission spot diameter of 200 μm. Only when the laser beam irradiated the thermocouple junction area and its surrounding area was there a non-noise voltage output between the two ends of the thermocouple. The output voltage of each thermocouple in the array were monitored in laser fiber’s positioning operation. The 3D locator bound with the optical fiber was adjusted slowly until an output voltage of the thermocouples was produced, indicating that the laser beam was irradiating on or near the junction area. Later, we fine-tuned the position of the fiber in a selected direction (X-axis or Y-axis) as shown as the blue line in Figure 2b, until the voltage output of the thermocouple reached the maximum value. At this time, the laser beam irradiated site 3 in Figure 2b. Then, we continued to fine-tune the position of the fiber in another direction, vertical to the former direction, shown as the orange line in Figure 2b, until the voltage output reached the maximum value again. At this time, the laser beam was accurately irradiated on the junction area of the thermocouple, at site 6 in Figure 2b. The precise positioning method is illustrated in Figure 2b,c.

### 3.2. Evaluation of the Photothermal Effect in Micron-Sized Lasers Interacting with the Pd/Cr Thermocouple

The photothermal effects of the pulsed micron-sized lasers with different wavelengths (450 nm, 525 nm, 638 nm, 810 nm, 980 nm, and 1064 nm) and different pulse frequency (mainly 50 Hz, 100 Hz, 300 Hz, 400 Hz, 500 Hz, 600 Hz, 800 Hz, and 1000 Hz) were evaluated using the homemade real-time temperature measurement system. Previous experimental results showed that a laser with a pulse width of 200 μs could cause neuronal excitement; therefore, the widths of the pulsed lasers in this work were all fixed at 200 μs.

**Figure 3:**
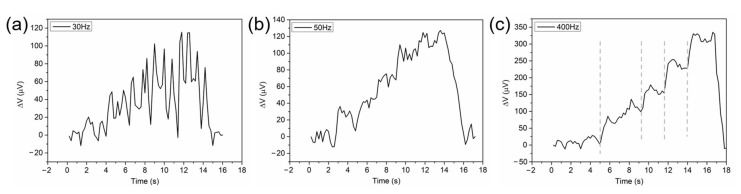
The output voltage curve versus peak value at different pulse frequencies: (**a**) 30 Hz, (**b**) 50 Hz, and (**c**) 400 Hz. The dashed gray lines represent the moments when the power of the laser is increased.

In the preliminary experiment, we observed the voltage output of the thermocouple, which represents the temperature change at the junction of thermocouples and further reflects the photothermal effect, as the peak power of the pulsed laser gradually increased. The experimental results illustrated in Figure 3 showed that when the repetition frequency of the pulsed laser was below 50 Hz, the output voltage curve behaved as many individual spikes, in which the peak value of the temperature change gradually increased with the peak power of the lasers, as shown in Figure 3a. This is because the heat irradiated on the Pd/Cr thin film thermocouple by a single pulse was quickly dissipated when the interval time between two adjacent laser pulses was too long, i.e., the frequency is very low. When the pulse frequency was larger than 100 Hz, the voltage output curve versus time drawn by the LabVIEW program displayed stepping up with the increase in the power peak of the laser beam, as shown in Figure 3c. Furthermore, the output voltage was maintained in a relatively stable range when the peak power was constant. This phenomenon makes the temperature measurement reliable for evaluating photothermal effects of lasers–Pd/Cr interactions possible. As a result, pulse frequencies with a value of ≥50 Hz were chosen in this work, and the average temperature rise on the stairs in the voltage output curve was taken as the temperature change under the corresponding laser irradiation.

**Figure 4:**
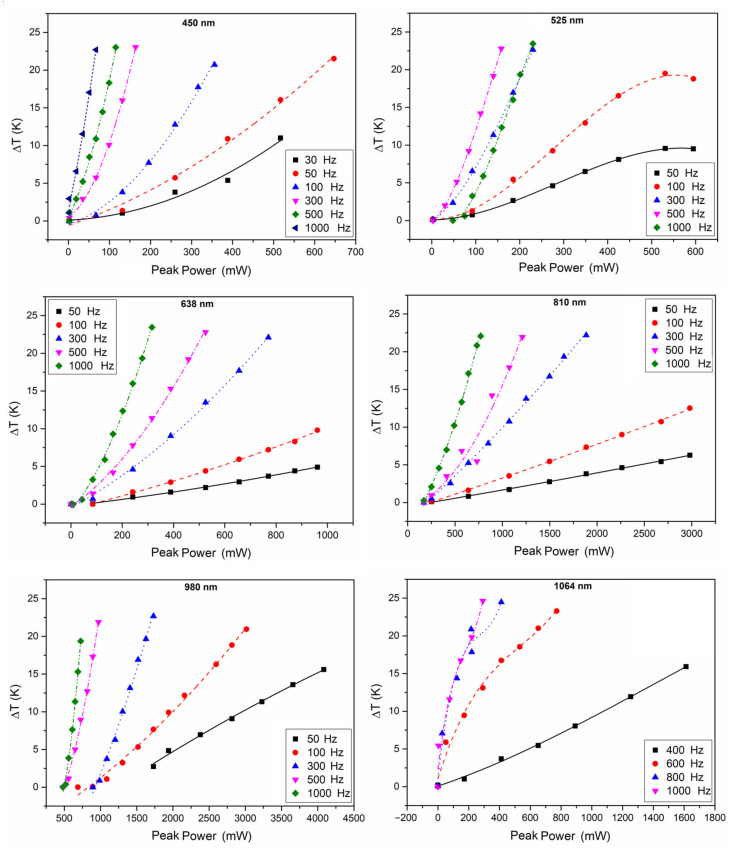
The relationship between the temperature rise owing to photothermal effect and the peak power of pulsed lasers with different wavelengths and different pulse frequencies.

The relationships between the temperature rise of the Pd/Cr thin film thermocouple junction and the peak power of lasers with different wavelengths (450 nm, 525 nm, 638 nm, 810 nm, 980 nm, and 1064 nm) are all plotted in Figure 4. For lasers with the same frequency, the local temperature increased rapidly with the pulse frequency when the peak power was fixed, which is a conceivable result. As the frequency increased, the laser energy irradiated on the thermocouple per second increased by a corresponding multiple, resulting in increasing the temperature of the thermocouple.

**Figure 5:**
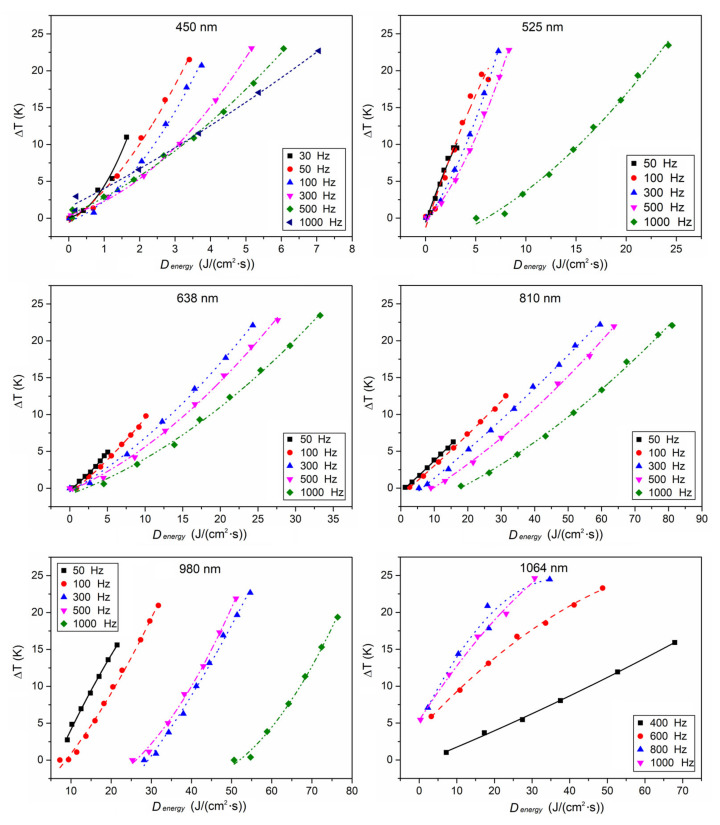
The relationship between the temperature rise (ΔT) resulting from the photothermal effect of laser–Pd/Cr thermocouple interactions and the average energy density as well as pulse frequencies for lasers with different wavelengths.

The relationships between the temperature rise and the average energy density of the lasers with different wavelengths are also plotted in Figure 5. The results show that the temperature rise is nearly linearly proportional to the average energy density of a laser when its pulse frequency is fixed, indicating that the ratio of photothermal effects to the total laser energy is relatively stable in the laser–Pd/Cr film interaction. Moreover, when the average energy density is the same, the local temperature change will decrease with the pulse frequency for the lasers with the wavelength range within 450–980 nm. This demonstrates that compared to the total irradiation duration, the laser intensity impacts more on the local temperature rise of thermocouples. However, for a laser with 1064 nm wavelength, the experimental result was the opposite; the local temperature change increased with the pulse frequency. This seems to indicate that compared with the lasers with a shorter wavelength, the strength of the photothermal effect is more irradiation time-dependent for the 1064 nm laser.

## 4. Discussion

### 4.1. Relationship between the Photothermal Effect of Laser–Thermocouple Interactions and the Wavelength

Due to differences in the laser wavelength, pulse frequency and peak power, the heat collected from the irradiation of focused laser beams with different light parameters by the same thermocouple is different. The above results show that the temperature rise of the Pd/Cr thermocouple caused by laser–Pd/Cr interactions is closely related to the peak power and pulse frequency of the laser. In order to explore the dependence of photothermal responses on wavelengths, the comparison of peak power required to increase the same temperature of the thermocouple junction area at the same pulse frequency, which is actually the required total energy, should be studied for lasers with different wavelengths. Therefore, we extracted the data of peak power required by the lasers at different pulse frequencies of 300 Hz, 500 Hz, and 1000 Hz to raise the junction temperature by 15 °C and 20 °C. As shown in Table 1, at a fixed pulse frequency, the peak power required for the same temperature change increased with the laser wavelength in the range of 450–810/980 nm, whereas it decreased with the wavelength for lasers of 810, 980 and 1064 nm. The results mean that the photothermal effects of lasers with a wavelength of 810 nm and 980 nm seem the worst, because they need more energy to increase the same temperature of the Pd/Cr thermocouples. The largest values of the required peak powers at each pulse frequency in Table 1 are highlighted in bold.

**Figure 6:**
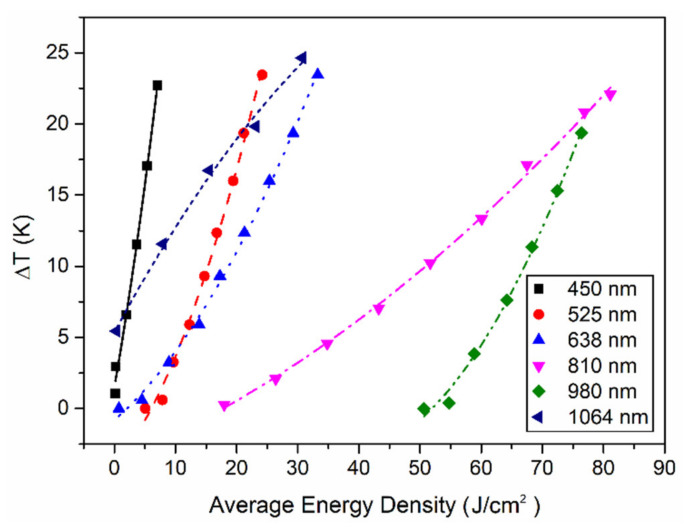
The temperature rises versus the average energy density of the lasers with different wavelengths at the pulse frequency of 500 Hz.

To compare the photothermal effects of lasers with different wavelengths more directly, we analyzed the relationship between the temperature rise caused by laser–Pd/Cr metal film interactions and the laser energy at a pulse frequency of 500 Hz, as shown in Figure 6. For lasers with wavelengths below 810 or 980 nm, the temperature rise is inversely proportional to the wavelength, consistent with Table 1. In summary, in the visible light band (450–810 nm), the shorter the wavelength of the laser, the better it demonstrates the photothermal effect; however, in the infrared light band (980–1064 nm), it seems that the longer the wavelength, the better the photothermal effect. This photothermal effect law in laser–Pd/Cr metal film interactions is unexpectedly consistent with that in laser–biological tissue interactions [38,39]. For example, the photothermal effects of lasers with different wavelengths were reported in 2018, where 532 and 1470 nm induced higher temperatures for liver tissues than those induced by 808 and 980 nm [38]. Interestingly, it is well known that the composition and morphology of biological tissue is quite different from metal film. The mechanism of the similar change trend between them needs to be further studied in the future.

### 4.2. Feasibility of Applying this System to Photothermal Evaluation

The process of thermocouple temperature sensor measuring the temperature rise generated by laser irradiation can be divided into two stages from the microscopic viewpoint. First, the electron, A, in the metal junction of thermocouples jumps to the excited state A * after absorbing the photon with an energy of *hν*. Later, the excited electron A * collides inelastically with the medium M in the surroundings, which causes A* to deactivate and increases the kinetic energy of M at the same time. This process of energy conversion from lasers into the kinetic energy of metal molecules leads to a rise in the temperature of Pd/Cr metal junctions. These two stages can be expressed as an absorption stage, A + *hν*→A *, and a deactivation stage, A * + M (E_k_) →A + M (E_k_ + ΔE_k_).

The dependence of photothermal effects in laser–Pd/Cr interactions on wavelengths can be well explained with the above microscopic analysis. The shorter the wavelength, the higher the photon energy of the laser, which benefits the higher energy of metal electrons. Thus, the kinetic energy of the metal medium M will increase more via inelastic collisions with excited electrons. Temperature reflects the average thermal kinetic energy of microscopic metal particles. As a result, under the same irradiation energy, the higher temperature of the metal is caused by light with shorter wavelengths in the visible band. As for the unusual high photothermal effect of infrared band lasers, further study is needed. Another reason for the wavelength dependence of photothermal effects in laser–Pd/Cr interactions is that the absorption rate of metal to light varies with the wavelength [40,41], which also contributes to the final displayed total law of photothermal effects.

This work reflects the high absorption of laser radiation by the metal junction of the Pd/Cr thermocouple. It is conceivable that when the thermocouple device is used to evaluate the photothermal effect of laser–tissue interactions, the heat generated by the thermocouple itself due to the irradiation of the attenuated laser after penetrating tissues may interfere with the measurement results. Fabrice Manns’s team in Miami University reported that when the commercial stainless-steel thermocouple was close to the laser beam, the temperature of the thermocouple itself will rise by nearly 20 °C [42], which will introduce a significant overestimation on the actual temperature of the measured tissues.

To reduce this interference, the size of the thermocouple junction for detecting temperature used in this study was designed at the sub-micron scale, 3 μm × 3 μm × 100 nm. Thus, the temperature rise caused by the metal thermocouple itself should be much weaker. In addition, the thin film thermocouple employs the contact-type method to detect the tissues’ temperature in the research of laser–tissue interactions, i.e., the examined tissues are located between the laser beam and the thermocouple sensor. The intensity of the laser penetrating the tissue and irradiating on the thermocouple junction will remain too low to lead to obvious impacts. An early study confirmed this conclusion indirectly [29]. Therefore, we infer that the system based on a Pr/Cr thin film thermocouple can truly reflect the temperature change of a tissue being measured in photothermal evaluations, especially when combined with the obtained background reference data.

Although lacking biological experiments, experiments on thermocouple irradiated by lasers are sufficient to verify the feasibility of the homemade system in evaluating the photothermal effects of micron-sized lasers interacting with other tissues. Referring to the obtained basic data, the mathematical model of photothermal effect in laser–tissue interactions can be established using this system. In the future, this system can be applied in laser–neuron interaction studies to quantitatively evaluate the role of photothermal effects in stimulating neuronal excitement, which is beneficial for clarifying the response mechanism of auditory nerves under laser stimulation, and to promote the study of optical cochlear implants.

## 5. Conclusions

Combining a micron-scale Pd/Cr thin film thermocouple array, precise 3D position module and real-time data processing program, we have developed a stable, reliable and real-time temperature measuring system to quantitatively evaluate the photothermal effects of micron-sized lasers interacting with other tissues. Using this system made in-house, we studied the influencing factors of the photothermal effect of micron-sized pulsed lasers, such as wavelengths, peak power and pulse frequencies, when they directly irradiated the thermocouples without other tissues. The measurement results showed that when the wavelength and pulse frequency were fixed, the temperature measured by the thermocouple changed linearly with the energy density or peak power, indicating the stability of the light-to-heat ratio. The photothermal effect behavior was negatively related to the laser wavelength in the visible light band, but positively in the infrared light band. This work not only provides a universal measuring system for photothermal evaluations in the interaction between micron-sized lasers and other tissues in the future, but also provides basic reference data for the construction of a photothermal effect model.

## Figures and Tables

**Table 1:** The peak power required to increase the temperature of the thermocouple junction area for different pulse frequencies for the pulsed lasers with different wavelengths.

	Peak Power Required for a Temperature Increase of 15 °C			Peak Power Required for a Temperature Increase of 20 °C		
Wavelength (nm)	Peak Power (mW)			Peak Power (mW)		
	300 Hz	500 Hz	1000 Hz	300 Hz	500 Hz	1000 Hz
450	126.1	84.6	45.3	150.3	104. 9	59.6
525	169.4	116.6	175.9	209.3	143.4	206.9
638	578.2	389.5	234.1	714.5	475.1	283.2
810	1370.9	981.5	607.3	1717.8	1144.9	717.8
980	1479.5	855.2	686.5	1637.0	939.1	726.0
1064			118.2			222.8

## Data Availability

The data presented in this study are available on request from the corresponding author.
